# Long-term production of BDNF and NT-3 induced by A91-immunization after spinal cord injury

**DOI:** 10.1186/s12868-016-0267-6

**Published:** 2016-06-30

**Authors:** Susana Martiñón, Elisa García-Vences, Diana Toscano-Tejeida, Adrian Flores-Romero, Roxana Rodriguez-Barrera, Manuel Ferrusquia, Rolando E. Hernández-Muñoz, Antonio Ibarra

**Affiliations:** Facultad de Ciencias de la Salud, Centro de Investigación en Ciencias de la Salud (CICSA), Universidad Anáhuac México Norte, Huixquilucan, Estado de México Mexico; Centro de Investigación del Proyecto CAMINA A.C., Mexico, D.F. Mexico; Instituto Nacional de Psiquiatría “Ramón de la Fuente Muñiz”, Mexico, D.F. Mexico; Departamento de Biología Celular y Desarrollo, Instituto de Fisiología Celular, UNAM, Mexico, D.F. Mexico

**Keywords:** Protective autoimmunity, Paraplegia, Neurotrophic factors, Neural derived peptides, A91, Immunization

## Abstract

**Background:**

After spinal cord (SC)-injury, a non-modulated immune response contributes to the damage of neural tissue. Protective autoimmunity (PA) is a T cell mediated, neuroprotective response induced after SC-injury. Immunization with neural-derived peptides (INDP), such as A91, has shown to promote—in vitro—the production of neurotrophic factors. However, the production of these molecules has not been studied at the site of injury.

**Results:**

In order to evaluate these issues, we performed four experiments in adult female Sprague–Dawley rats. In the first one, brain derived neurotrophic factor (BDNF) and neurotrophin-3 (NT-3) concentrations were evaluated at the site of lesion 21 days after SC-injury. BDNF and NT-3 were significantly increased in INDP-treated animals. In the second experiment, proliferation of anti-A91 T cells was assessed at chronic stages of injury. In this case, we found a significant proliferation of these cells in animals subjected to SC-injury + INDP. In the third experiment, we explored the amount of BDNF and NT3 at the site of injury in the chronic phase of rats subjected to either SC-contusion (SCC; moderate or severe) or SC-transection (SCT; complete or incomplete). The animals were treated with INDP immediately after injury. Rats subjected to moderate contusion or incomplete SCT showed significantly higher levels of BDNF and NT-3 as compared to PBS-immunized ones. In rats with severe SCC and complete SCT, BDNF and NT-3 concentrations were barely detected. Finally, in the fourth experiment we assessed motor function recovery in INDP-treated rats with moderate SC-injury. Rats immunized with A91 showed a significantly higher motor recovery from the first week and up to 4 months after SC-injury.

**Conclusions:**

The results of this study suggest that PA boosted by immunization with A91 after moderate SC-injury can exert its benefits even at chronic stages, as shown by long-term production of BDNF and NT-3 and a substantial improvement in motor recovery.

## Background

Spinal cord (SC)-injury triggers a cascade of events that include an important local inflammatory response at the site of injury [1]. Inflammation, although commonly elicited in order to achieve repair of injured tissues, has deleterious effects that lead to increased neuronal loss and poor functional recovery after SC-injury [2]. Therefore, regulation of this phenomenon after injury is of imperative importance in order to limit these destructive effects.

Protective autoimmunity is a physiological T-cell-dependent, anti-inflammatory and neuroprotective immune response to central nervous system (CNS) trauma [3, 4]. In fact, studies have shown that autoimmune T cells directed against CNS myelin basic protein (MBP) promote recovery after contusion of the SC [5].

One way of boosting protective autoimmunity is through active immunization with non-encephalitogenic peptides. A91 is a peptide derived from MBP (sequence 87–99) in which the lysine residue at position 91 has been replaced with alanine. Active immunization with A91 induces the proliferation of CNS-antigen-specific T-cells (in this case, anti A91 T-cells). These cells, instead of having deleterious inflammatory effects, exert protective actions, which promote neuroprotection through the reduction of nitric oxide and lipid peroxidation [6, 7].

Several studies from our laboratory have shown that immunization with A91, alone or in combination with other strategies, improves functional recovery after SC-injury [8, 9]. Nevertheless, in animals with severe SC-injury, these beneficial effects are not observed [9]. Additionally, we have found that active immunization with A91 induces a Th2 phenotype expression [9], these cells were found capable of releasing significant amounts of brain-derived neurotrophic factor (BDNF) when activated in vitro with A91-peptide. This finding could explain, at least in part, the beneficial effects on functional recovery after SC-injury observed in rats immunized with A91 [9]. In the light of these findings we decided now to explore whether A91-peptide immunization is capable of increasing BDNF and/or neurotrophin-3 (NT-3) concentrations at the site of injury shortly after SC-injury and if this production is still present at chronic stages.

## Methods

### Experimental design

Sample size for each experiment was calculated using an alpha of 0.05 and beta of 0.20. All animals were randomized to each experiment and basal statistical analysis of weight and age yielded no statistical significance between experimental sets.

Four experiments were performed. In the first one, we explored whether A91 immunization is capable of inducing BDNF and NT3 at the site of lesion, specifically shortly after SC-injury. In the second experiment, we study if A91 immunization induced a T-cell response that could be observed up to the chronic stage of injury (4 months). In the third experiment, we investigated whether A91 immunization induces a long-term (after 4 months) production of BDNF and NT-3 at the site of injury. In this case, rats were subjected to SC-injury as follows: a first group of rats was subjected to a moderate or severe contusion. In a second group, rats were subjected either to hemisection or a complete SC-transection. Each experiment (experiments 1–3) was performed by triplicate. Finally, in the fourth experiment the motor recovery of animals with moderate SC-injury was investigated.

### Animals

Adult female Sprague–Dawley rats (13–14 weeks old, 200–220 g) were supplied by the Animal Breeding Center of Proyecto Camina A. C. Efforts were made to minimize the number and suffering of animals used for this project.

### Spinal cord injury

Rats were anesthetized by intramuscular injection of ketamine (80 mg/kg; PISA Laboratories, Mexico City, Mexico) and xylazine (12.5 mg/kg; Bayer Laboratories, Mexico City, Mexico). After anesthesia induction, a laminectomy and exposure of the spinal cord was performed at T9. Rats were subjected to spinal cord contusion (SCC) or spinal cord transection (SCT). For SCC, a 10-g rod was dropped onto the exposed spinal cord from a height of 25 or 50 mm for a moderate or severe contusion, respectively, using the NYU impactor (NYU, New York). This device is capable of inflicting a well-calibrated contusive injury of the SC [10]. For SCT, the dura mater was dissected and separated from the spinal cord with a 30-gauge needle. Complete transection was performed using iridectomy scissors. Accuracy of the injury was visually verified by passing a micro-hook through the internal contour of the dura. For incomplete transection, approximately 50 % of the dorsal spinal cord was transversely cut sliding a straight-edged scalpel blade through the spinal cord. After injury, the aponeurotic plane was sutured with poliglycolic acid and the skin, with nylon thread.

### Animal care

Animals were matched for age and weight in each experiment and housed in pairs in a light- and temperature-controlled room. To minimize stress, animals were handled daily at least once a day 7 days prior to the surgical procedure.

Sterile bedding and filtered water was replaced daily. Bladder emptying was performed by manual expression three times a day until automatic voidance was regained. During the first day after injury, the animals received a course of enrofloxacine (Marvel, Mexico City, Mexico) in their drinking water at an approximate dose of 64 mg/kg/day. All rats were carefully monitored for evidence of postsurgical complications. Animals with signs of infections were excluded from the study.

### Antigen (A91 peptide)

A91 peptide was derived from the encephalitogenic sequence of myelin basic protein (MBP; amino acids 87–99). Non-encephalitogenicity was obtained by replacing the lysine residue at position 91 with alanine. The modified peptide was purchased form Invitrogen Life Technologies (San Diego CA, USA). Reverse- phase HPLC confirmed that the purity of the A91 peptide was >95 %.

### Active immunization

Rats were immunized subcutaneously at the base of the tail with 150 μg of A91 in phosphate buffered saline (PBS), emulsified in an equal volume of complete Freund’s adjuvant (CFA) containing 0.5 mg/ml *Mycobacterium tuberculosis* (Sigma, St. Louis MO). Immunization was performed within a 60 min frame after injury.

### T cell proliferation

Cells were pooled from excised inguinal lymph nodes 4 months after SC-injury. The cells were cultured in quintuplicate flat-bottomed wells in 0.2 ml of RPMI-1640 medium (Gibco, New York) supplemented with 10 % fetal bovine serum (Gibco, New York) on a 96—well microtiter plate. Cells (2.5 × 10^5^ cells per well) were cultured 72 h in antigen-free medium or together with A91 (10 μg/ml), ovalbumin (OVA; 10 μg/ml; Sigma), or concanavalin-A (ConA; 10 μg/ml; Sigma St. Louis MO) at 37 °C in 5 % CO_2_. After two washes with RPMI-1640, cells were labeled with carboxyfluorescein diester anime (CFSE) (Molecular Probes). CFSE-labeled cells divide and its progeny are endowed with half the number of carboxyfluorescein-tagged molecules, thus each cell division can be assessed by measuring the corresponding decrease in cell fluorescence. 5 μl of CFSE at a final concentration of 1 μM were rapidly dispensed into the cell suspension insuring a homogeneous labeling. Cells were incubated for 24 h at 37 °C. Staining was halted by the addition of an equal volume of fetal bovine serum. The proliferative response was determined by flow cytometry. Cells were also stained with phycoerythrin-labeled anti-CD4 monoclonal antibodies (BD Pharmigen, San Diego, CA); unstained cells were used as controls. Cells stained with CFSE and CD4 were analyzed.

For analysis, the area of lymphocytes was selected based on the light scattering characteristics (size/granularity) of these cells. Afterwards, the area of CD4+ cells was selected and analyzed for CFSE fluorescence. Mean fluorescence intensity data was obtained from fluorescence histograms to evaluate the fractions of T cells that have completed a given number of divisions. Ten thousand events were collected for each sample of a FACSCAlibur flow cytometer (BD Bioscence, Mountain View, CA) and analyzed using CellQuest Pro software (BD Bioscences). The stimulation index (SI) was calculated by dividing the mean percentage of proliferation in experimental wells by the mean percentage of proliferation the corresponding control wells (cells cultured in antigen-free medium).

### BDNF and NT3 analysis

After lethal pentobarbital injection, SC samples (2.5 cm including the site of injury) were rapidly excised. The tissue samples were weighed and snap frozen in liquid nitrogen prior to storage at −70 °C. Within 2 weeks of freezing, tissue samples were homogenized in ice cold homogenization buffer consisting of 100 mM Tris/Hcl, pH 7, 2 % bovine serum albumin (BSA), 1 M NaCl, 4 mM EDTA, 2 % Triton X-100, 0.1 % NaN_3_, and the following protease inhibitors: 5 µg/mL aprotinin, 0.5 µg/mL antipain, 157 µg/mL benzamidine, 0.1 µg/mL pepstatin A and 17 µg/mL phenylmethyl-sulphonyl fluoride. Homogenates were prepared in approximately 20 volumes of the homogenization buffer to tissue-wet weight. The homogenates were centrifuged at 14,000×*g* for 30 min. The resulting supernatants were divided into two equal samples and used for the BDNF and NT-3 assays. The samples were analyzed by triplicate and following the instructions of the ChemiKine™ BDNF and NT-3 Sandwich ELISA Kit (Millipore, USA). Absorbance was measured in a microplate spectrophotometer at a 450 nm wavelength (MultiSkan, Thermo Scientific, Finland).

### Assessment of motor recovery

Behavioral recovery was assessed every week after spinal cord contusion using the Basso, Beattie and Bresnahan (BBB) open-field test of locomotor ability [11]. Three separate blinded observers evaluated all animals and the average of the three scores was used.

### Statistical analysis

Data was analyzed using the GraphPad Prism 3.0 software and presented as mean ± standard deviation (SD). The proliferative response was evaluated using the Student’s t-test. BDNF and NT-3 levels were analyzed using a Mann-Whitey U test. Motor recovery was evaluated using a two-way ANOVA for repeated measures. Differences of p ≤ 0.05 were considered statistically significant.

## Results

### Production of BDNF and NT-3 is increased in the site of injury after A91-immunization

**Fig. 1:**
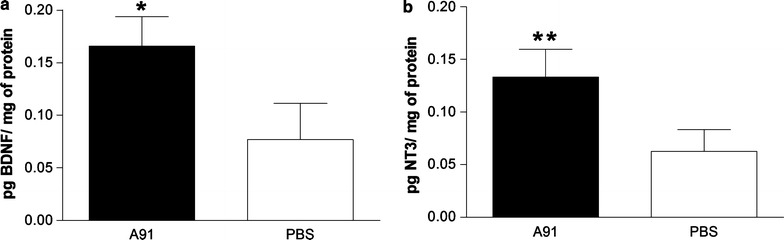
A91-immunization increases the levels of BDNF and NT-3 at the site of injury. Twenty-one days after injury the levels of this molecules were significantly higher in A91-immunized rats than those observed in PBS-immunized ones. *Bars* represent the mean ± SD of 5 rats. This is one representative of 3 experiments. *Different from PBS, p = 0.002; Mann–Whitney U test; **Different from PBS, p = 0.03, Mann–Whitney U test

In a previous work, we reported that anti-A91 T cells from SC injured rats are capable of producing BDNF after an in vitro challenge with A91-peptide [9]. In line with this, we investigated now if A91-immunization is capable of increasing the levels of BDNF and/or NT-3 at the site of injury. For this purpose ten rats were subjected to a moderate contusion and immediately immunized with a single dose of A91 (n = 5) or PBS (n = 5). Twenty-one days after SC-injury (time to ensure the activation of A91-reactive T-cell in this experimental model) we determined the levels of BDNF or NT-3 at the site of lesion. As can be seen in Fig. 1, A91-immunization induced a significant increase in both BDNF and NT-3 molecules. The levels of BDNF were of 0.165 ± 0.01 (mean ± SD) in A91-immunized animals while the ones observed in PBS-immunized ones were of 0.076 ± 0.02 (p = 0.002; Mann–Whitney U test, Fig. 1a). In the same way, NT-3 concentrations in rats receiving A91-immunization were significantly higher (0.133 ± 0.02) than those presented by PBS-immunized animals (0.062 ± 0.01; p = 0.03, Mann–Whitney U test, see Fig. 1b).

### Long-term immune response and production of BDNF and NT-3 in A91-immunized rats

**Fig. 2:**
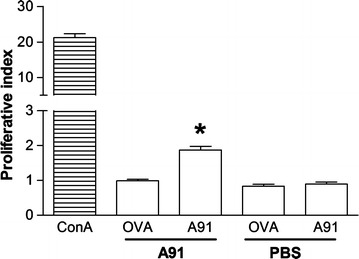
Immunization with A91-peptide elicits an immune response that is detected up to 4 months after SC injury. Anti-A91 response was significantly higher in rats immunized with A91 relative to those immunized only with PBS. *Bars* represent the mean ± SD of 5 rats. This is one representative of 3 experiments. *Different from PBS-immunized rats, p = 0.001, Student t test. *OVA* ovalbumin, *ConA* concanavalin A

Based on the fact that SC-injury induces a chronic response against neural antigens [12] and considering that neural-derived peptides are proficient to induce an immune reaction [8] we decided to explore if A91-immunization is capable of inducing a long-term specific T-cell response that could maintain or increase the production of neurotrophic factors, even in the chronic phase of injury. In order to elucidate this issue, in a first step, we proceeded to determine the proliferative response of anti-A91 T cells in rats subjected to SC-injury. Thus, rats were subjected to a moderate contusion and then immediately immunized with A91 peptide (n = 5) or only PBS (n = 5). Proliferation of anti-A91 cells was assessed 4 months after SC-injury. Figure 2 shows that A91-immunization induced a long-term response. The stimulation index observed in A91-immunized rats was significantly higher (1.87 ± 0.09, mean ± SD) as compared to the one presented by PBS immunized animals (0.89 ± 0.05; p = 0.001, Student t test).

**Fig. 3:**
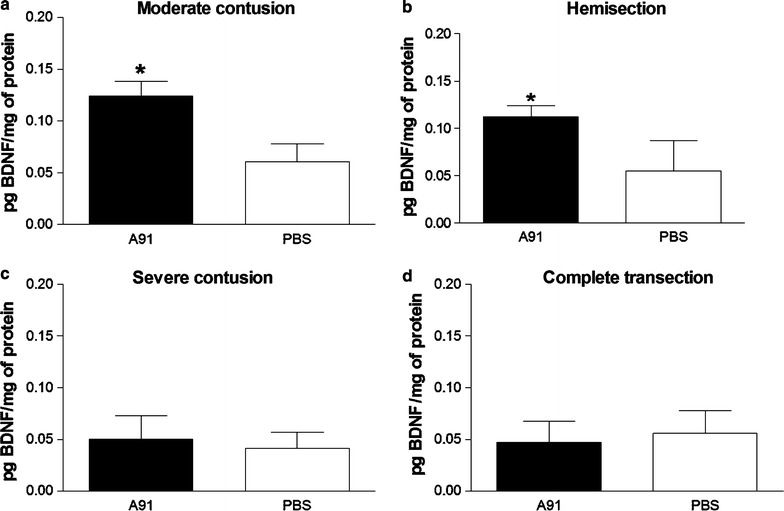
BDNF concentrations 4 months after SC injury. The levels of BDNF in A91-immunized rats were significantly higher only in rats with moderate contusion (**a**) or hemisection (**b**). In rats with severe contusion (**c**) or complete transection (**d**) A91-immunization did not increase BDNF levels. *Bars* represent the mean ± SD of 5 rats. This is one representative of 3 experiments. *Different from PBS, p = 0.004; Mann–Whitney U test; **Different from PBS, p = 0.005, Mann–Whitney U test

**Fig. 4:**
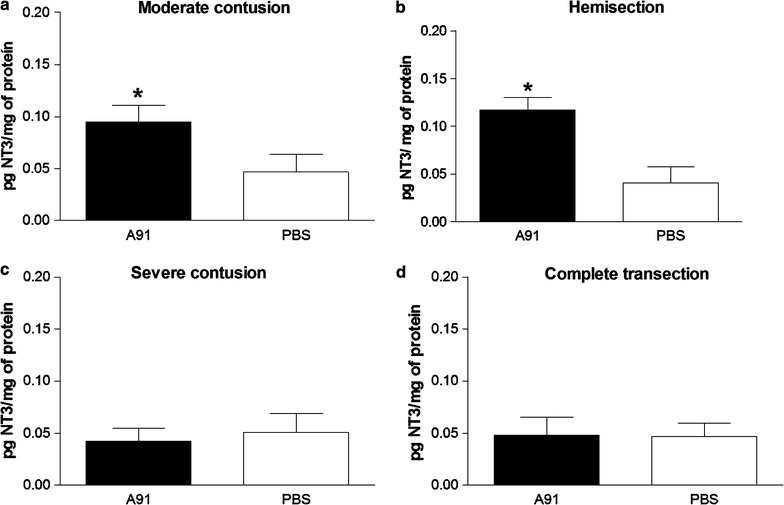
NT3 levels 4 months after SC injury. NT3 concentrations were increased only in rats with moderate contusion (**a**) or hemisection (**b**). A91-immunization failed to increase the levels of NT3 in severely contused (**c**) and transected (**d**) rats. *Bars* represent the mean ± SD of 5 rats. This is one representative of 3 experiments. *Different from PBS, p = 0.008; Mann–Whitney U test; **Different from PBS, p = 0.005, Mann–Whitney U test

In view of the above results, we then explored the levels of BDNF and NT-3 molecules at the site of lesion of rats with SC-injury in the chronic stage (4 months after SC-injury). In order to attain a more comprehensive study, we designed a broad experiment where other different models of SC-injury were included. In this way, ten rats were subjected to a moderate (n = 5) or a severe (n = 5) contusion. In the same experiment, other ten rats were subjected to hemisection (n = 5) or a complete SC-transection (n = 5). BDNF and NT-3 levels were determined by ELISA assay and thus were compared among all groups. This experiment was performed by triplicate. Figures 3 and 4 show that A91 immunization elicited the production of BDNF and NT3 in chronic stages of injury; however, this effect was not observed in all SC-injury models. Rats immunized with A91–peptide and subjected to moderate contusion (Fig. 3a) or hemisection (Fig. 3b) presented higher levels of BDNF (0.12 ± 0.01 and, 0.11 ± 0.02 respectively, mean ± SD) as compared to PBS-immunized ones (0.06 ± 0.01 and 0.05 ± 0.03 respectively; p = 0.004, Mann–Whitney U test). In contrast, the levels of this molecule were barely detected in animals with severe contusion (A91: 0.05 ± 0.02 vs PBS: 0.04 ± 0.01; p > 0.05, Mann–Whitney U test, Fig. 3c) or complete transection (A91: 0.04 ± 0.02 vs PBS:0.06 ± 0.01 p > 0.05, Mann–Whitney U test; Fig. 3d). In the case of NT3, the results were quite similar. A significant increase was observed in A91-immunized rats that were subjected to moderate contusion (0.09 ± 0.01, Fig. 4a) or hemisection (0.11 ± 0.01, Fig. 4b) in comparison to those immunized only with PBS (0.05 ± 0.02 and 0.04 ± 0.01 moderate contusion and hemisection respectively, p = 0.005, Mann–Whitney U test). A91-immunization did not induce any significant increase in NT-3 levels when rats were subjected either to severe contusion (Fig. 4c, A91: 0.04 ± 0.01 vs PBS: 0.05 ± 0.02.; p > 0.05, Mann–Whitney U test) or complete transection (Fig. 4d, A91: 0.05 ± 0.02 vs PBS 0.05 ± 0.01; p > 0.05, Mann–Whitney U test). In this rats NT-3 concentrations were also barely detected.

### A91-immunization maintained and improved motor performance in chronic stages of injury

**Fig. 5:**
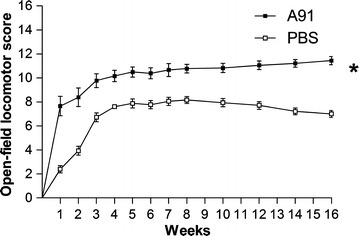
Motor recovery of rats with moderate SC-contusion. Animals were treated either with A91 or PBS. A91-immunization improved motor performance. *Different from PBS group (p = 0.01, two-way ANOVA for repeated measures). *Each point* represents the mean ± SD of 10 rats

In order to explore if the microenvironment induced by A91-immunization improves locomotor performance at chronic stage of SC-injury, we assessed motor function in rats with moderate contusion treated with either A91 (n = 10) or PBS (n = 10) immunization immediately after SC-injury. Evaluations were performed each week up to 4 months after injury using the BBB scale. Figure 5 shows that A91-immunized rats presented higher BBB scores than those immunized only with PBS. From the beginning (1 week after SC-injury), A91-immunization induced a significant increase in motor recovery (6.3 ± 0.2; mean ± SD) compared with PBS-treatment (2.4 ± 0.3). Two months after injury, A91-immunized rats continued showing a better BBB score (10.8 ± 0.2) when compared to PBS-treated ones (8.1 ± 0.3). At the end of the study, A91-immunization promoted an even better motor performance (11.4 ± 0.3) compared to the one observed for the same group 2 months before (p = 0.02 Wilcoxon signed rank test). In contrast, animals immunized with PBS, showed a significant decline in the BBB score (7.0 ± 0.4) when compared to the one observed 2 months before (p = 0.03 Wilcoxon signed rank test).

## Discussion

Protective autoimmunity (PA) is a physiological and neuroprotective immune response mediated by T cells [4]. This innovative approach is in fact a T-cell-dependent reaction that is genetically determined since only in animals of EAE-resistant strains (but not of susceptible strains), the injury evokes an endogenous protective response [13]. According to previous studies, genetic differences originate a more intense inflammatory response in susceptible strains; a response that in nature, is detrimental instead of beneficial. Several studies have shown that PA is activated by the interaction of T cells with neural constituents present at the site of lesion after SC-injury [3, 14, 15]. T cells interact with macrophages and resident microglia in an attempt to induce a protective response [3, 15]; however, the non-permissive microenvironment developed after injury leads to its failure and PA is not sufficiently capable of protecting SC tissue [15, 16]. Therefore, strategies to improve the effect of PA have been addressed.

A way of enhancing PA is through active immunization with neural-derived peptides such as A91. Neural-derived peptides interact with the T cell receptor (TCR) in a manner that modulates cytokine production [17, 18]. Additionally, these peptides promote T cell differentiation into a Th2 phenotype capable of secreting IL-4 and IL-10 [9], cytokines that down-regulate the expression of NO [19]. Moreover, A91 induced PA has the ability to ameliorate neurotoxicity mainly through lipid peroxidation inhibition [7], decrease in glutamate toxicity [20, 21], reduction of apoptosis triggered by SC-injury [22] and amelioration of other neurotoxic processes [6, 23]. Collectively, these findings provide evidence to support the contention that PA is more a beneficial phenomenon than a detrimental one; however; there is still controversy about the idea of recognizing an autoreactive response as a protective or restorative phenomenon. In this respect, previous studies have shown that neural-reactive T cells of patients with SC-injury are in fact, similar to those found in patients with Multiple Sclerosis (MS). In both cases, T cells recognize the same antigenic regions [24]. Nevertheless, the difference in the cytokine production between the two reactive T cells suggests that those derived from MS patients have a much higher inflammatory potential.

In light of this scenario we could hypothesize that autoreactive responses could behave either as a harmful or a beneficial phenomenon. Autoreactivity is influenced by several factors (e.g. genetic predisposition) that could induce the development of an uncontrolled immune response that attacks the CNS. This is what we observe in MS. On the other hand, the modulation of autoreactive responses has shown to improve neurological function in MS and other neurodegenerative diseases [8, 24, 25]. Accordingly, if the reaction is immediately modulated (by immunizing with neural-derived antigens), the autoreactive response is capable of providing beneficial effects.

Results from previous studies conducted in our laboratory have shown that immunization with A91 confers a significant improvement in motor function compared to controls after SC-injury. Regarding the mechanisms underlying PA, we demonstrated that A91 immunization induces a specific anti-A91 response that is capable of producing—in vitro—brain derived neurotrophic factor (BDNF), a molecule that induces axonal regeneration and confers motor function improvement after SC-injury. Some studies have also shown that BDNF and another neurotrophic factor, NT-3, prevent neuronal apoptosis [26, 27] and promote axonal sprouting and regeneration [28–30].

In the present study we demonstrate that BDNF and NT-3 production is increased at the site of injury after active immunization with A91. Moreover, 4 months after injury, immunized rats presented a significant response against A91-peptide and a concomitant production of BDNF and NT-3. These observations shed light onto beneficial effects of PA, even at chronic stages.

Previous studies have already reported the presence of immune cells at chronic phases of injury. For instance, Guizar-Sahagún et al. [31] reported the chronic presence of inflammatory cells (especially macrophages and lymphocytes) at the lesion site and nearby areas, even as late as 364 days post-injury. In the same way, Beck and colleagues reported cellular inflammation up to 180 days after SC-injury [32]. In their investigation they also showed that the presence of immune cells at this stage has a reparative function since it avoids neurological impairment. In the light of this finding, the presence of autoreactive responses against neural constituents in the late stages of SC-injury or traumatic brain injury (TBI) has also been documented and correlated with beneficial consequences [12, 33]. In the present work we showed that, even 4 months after A91 immunization, proliferation of anti-A91 T cells is still present. The mechanisms that underlie the presence of this response at chronic stages should be the aim of further investigations. Nevertheless, we can hypothesize that its presence is the result of two possible events: (1) anti-A91 T cells are locally cross-reacting with MBP [34], a molecule that could be presented by antigen presenting cells (APC) at the lesion site; and (2) once activated, T lymphocytes differentiate into memory cells that survive for a long time [35]. Therefore, immunization with A91-peptide could generate memory anti-A91 T cells that, along with APC in the site of injury, could be perpetuating the anti-A91 response. These issues should be further analyzed.

The results of the present work support A91 immunization as a plausible agent to be administered soon after SC-injury in order to promote beneficial mechanisms that can improve functional recovery even at chronic phases of injury. It is important to emphasize the fact that a single A91 administration could provide patients with a long-term neurotrophic factor source, providing concomitant therapies (such as physical rehabilitation) a propitious microenvironment for further motor recovery. The presence of neurotrophic factors in chronic stages of injury opens the possibility for restorative phenomena like neurogenesis, axonal sprouting and other reparative mechanisms, to maintain or even improve the motor performance observed in individuals with CNS injury [12, 33]. In line with this, our results show that rats receiving A91 immunization presented an improved motor performance that was maintained over time as compared to those treated with PBS only. This is an important finding that merits further investigation.

Finally, another topic to be addressed from this work is the absence of BDNF and NT-3 molecules after severe injuries. Previous investigations from our laboratory had shown that PA is not present after severe SCI [9]. In the present study, we investigated if BDNF and NT-3 are present at the site of injury in different SC-injury models. Our results show that PA and neurotrophic factor production after immunization with A91 is only present after moderate SCC and hemisection and that BDNF and NT-3 concentrations were barely present after severe SCC and complete transection. After SC-injury, a cascade of events that begin with the production of an autonomic discharge is unleashed. As a response to trauma, there is secretion of stress molecules, such as catecholamines and cortisol. Leukocytes have glucocorticoid and catecholamine receptors, and this hormone-cell interactions contribute to a state of immunodepression [36]. Additionally, prolonged exposure to catecholamines reduces the number of circulating T cells [37], producing an immunodeficiency called “SCI-induced immunodepression”. It is possible that this immunodepression limits the T cell capacity of processing and reacting towards a neural derived antigen, e.g. A91, thus preventing PA from occurring after immunization. Although the hypothesis of SCI-induced immunodepression impeding PA is plausible, the exact mechanisms and characteristics of SC-injury that are responsible for inhibiting the action of PA are still in need of further investigation.

## Conclusions

Immunization with A91 produces a chronic A91-reactive T cell response capable of promoting BDNF and NT-3 production during the chronic stages of SC-injury. Also, we observed that A91-immunized rats had an improved locomotor recovery that was maintained up to 4 months after the lesion. It is very likely that this improvement is the result of an enhanced microenvironment rich in neurotrophic factors induced by INDP.

The results from the present study bring hope to the clinical application of A91 as an immunomodulating therapeutic strategy. In the clinical prospect, nowadays there is no effective treatment after SC-injury. The only therapy that has been approved for human use is methylprednisolone, which has not shown consistent results on its efficacy in the long run [38].

A source of both BDNF and NT-3, such as PA boosted by A91, could confer benefits to patients suffering from SC-injury and increase their chances of functional recovery as well as offer an improvement in quality of life for them and their families.

## Abbreviations

BDNF: brain-derived neurotrophic factor
BSA: bovine serum albumin
CFA: complete Freund’s adjuvant
CFSE: carboxyfluorescein diester anime
CNS: central nervous system
INDP: immunization with neural-derived peptides
MBP: myelin basic protein
NT-3: neurotrophin-3
PA: protective autoimmunity
PBS: phosphate buffered saline
SC: spinal cord
SCC: spinal cord contusion
SCT: spinal cord transection
SD: standard deviation
TBI: traumatic brain injury
TCR: T cell receptor
